# Association between polymorphisms in XRCC1 gene and clinical outcomes of patients with lung cancer: a meta-analysis

**DOI:** 10.1186/1471-2407-12-71

**Published:** 2012-02-17

**Authors:** Zhigang Cui, Zhihua Yin, Xuelian Li, Wei Wu, Peng Guan, Baosen Zhou

**Affiliations:** 1 China Medical University, Shenyang 110001, PR China; 2 Department of Epidemiology, School of Public Health, China Medical University, Shenyang 110001, PR China; 3 Key Laboratory of Cancer Etiology and Intervention, University of Liaoning Province, Shenyang 110001, PR China; 4 Department of Epidemiology, Key Laboratory of Cancer Etiology and Intervention, China Medical University, University of Liaoning Province, No. 92, North Second Road, Heping District, Shenyang 110001, People's Republic of China

## Abstract

**Background:**

X-ray repair cross-complementing group 1 (XRCC1) protein plays an important role in the repair of DNA damage and adducts. Single nucleotide polymorphisms (SNPs) of *XRCC1 *are suspected to have some relationship with response to chemotherapy and overall survival of lung cancer. This meta-analysis aimed to summarize published data on the association between the commonest SNPs of *XRCC1 *(Arg194Trp, C > T, rs1799782 and Arg399Gln, G > A, rs25487) and clinical outcome of lung cancer patients.

**Methods:**

We retrieved the relevant articles from PubMed, EMBASE and the China National Knowledge Infrastructure (CNKI) databases. Studies were selected using specific inclusion and exclusion criteria. Primary outcomes included objective response (i.e., complete response + partial response vs. progressive disease + stable disease) and overall survival (OS). Odds ratio (OR) or hazard ratio (HR) with 95% confidence interval (CI) were estimated. All analyses were performed using the Stata software.

**Results:**

Twenty-two articles were included in the present analysis. *XRCC1 *Arg194Trp and Arg399Gln polymorphisms were significantly associated with response to treatment in lung cancer patients. Patients with C/T genotype, T/T genotype and minor variant T allele at Arg194Trp were more likely to respond to platinum-based chemotherapy compared with those with C/C genotype (C/T vs. C/C: OR, 2.54; 95%CI, 1.95-3.31; T/T vs. C/C: OR, 2.06; 95%CI, 1.39-3.06; C/T+T/T vs. C/C: OR, 2.42; 95% CI, 1.88-3.10). For *XRCC1 *Arg399Gln, G/A genotype, A/A genotype and minor variant A allele were associated with objective response in all patients (G/A vs. G/G: OR, 0.67; 95%CI, 0.50-0.90; A/A vs. G/G: OR, 0.43; 95%CI, 0.25-0.73; A/A+G/A vs. G/G: OR, 0.63; 95%CI, 0.49-0.83). Both G/A and A/A genotypes of *XRCC1 *Arg399Gln could influence overall survival of lung cancer patients (G/A vs. G/G: HR, 1.23; 95%CI, 1.06-1.44; A/A vs. G/G: HR, 2.03; 95%CI, 1.20-3.45). Interaction analysis suggested that compared with the patients carrying C/T+T/T genotype at *XRCC1 *194 and G/G genotype at *XRCC1 *399, the patients carrying 194 C/C and 399 G/A+A/A or 194 C/C and 399 G/G genotype showed much worse objective response.

**Conclusions:**

Genetic polymorphisms in *XRCC1 *gene might be associated with overall survival and response to platinum-based chemotherapy in lung cancer patients.

## Background

Lung cancer as a major public health problem represents the most common cancer, and more than a million people in the world die from the disease each year [1]. Despite the recent advances, the overall five-year survival rate of lung cancer is at only 15% in the United States and even lower in China [2]. Platinum is one of the most extensively used chemotherapeutic agents in lung cancer treatment, especially for the patients in advanced stages. However, the efficacy of platinum-based chemotherapy varied remarkably between different individuals, with a response rate from 26% to 60% in lung cancer patients [3]. Genetic factors are considered to influence the treatment effectiveness of lung cancer [4], thus affect the prognosis of patients. There are some molecular markers showing potential as therapeutic and prognostic indicators, but none could be used into clinical practice [5,6]. In these factors, DNA repair capacity (DRC) is an important one. The previous study has reported that effective host DRC may be associated with poorer survival in patients with non-small cell lung cancer (NSCLC) who are treated with chemotherapy [7]. It has been speculated that single nucleotide polymorphisms (SNPs) in DNA repair genes may change gene expression and activity, hence influence the effectiveness of cancer treatment and survival of patients [8]. X-ray repair cross-complementing group 1 (XRCC1) protein plays a central role in base excision repair (BER) pathway by interacting with other DNA repair proteins, giving the possibility that *XRCC1 *has some relationship with the response to therapy and the overall survival of lung cancer.

The *XRCC1 *gene was identified by its function to restore the DNA repair capacity in the Chinese hamster ovary mutant cell line EM9 [9] and interact with poly(ADP-ribose) polymerase and DNA ligase III in recognizing and rejoining DNA strand breaks, as well as with DNA polymerase β and apurinic/apyrimidinic endonuclease I [10-13]. The most extensively studied SNPs of *XRCC1 *gene are Arg399Gln (G > A, rs25487) and Arg194Trp (C > T, rs1799782), which have been reported to be associated with an altered DNA repair activity [14,15]. These polymorphisms that lead to amino acid changes might alter the efficiency of DNA repair and have functional significance. The functional effect of these polymorphisms is not clear, even though there are studies suggesting that amino acid substitutions at the evolutionary conserved regions can affect the protein ability [16]. Lamerdin et al. found that *XRCC1 *gene codon 194 was at a conserved residue in humans [17], suggesting the functional significance of this site. Theoretically, these polymorphisms could affect the response to cancer therapy through the removal of DNA adducts, in hence influence the overall survival of patients.

Some studies have reported the relationship between polymorphisms in *XRCC1 *gene and clinical outcome or overall survival of lung cancer patients [18-39], however the results were inconsistent. So we performed a systemic review and meta-analysis to assess the evidence about effects of *XRCC1 *SNPs on the efficacy of chemotherapy and overall survival in lung cancer patients.

## Methods

### Data sources

This meta-analysis focused on studies dealing with prognostic implication of *XRCC1 *SNPs in patients with lung cancer. We conducted this systematic review using a peer-reviewed, published protocol according to the guidelines of the Cochrane Collaboration. We retrieved the relevant articles using the following terms "*XRCC1 *or X-ray repair cross complementing protein 1" and "lung cancer or lung neoplasms" and "SNP or polymorphism" from PubMed, EMBASE and the China National Knowledge Infrastructure (CNKI) databases.

### Study selection and data extraction

**Figure 1:**
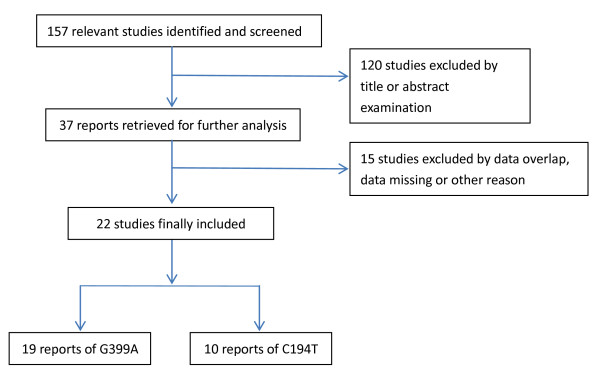
**Flow chart of the study selection process**.

Flow chart of the study selection process was shown in Figure 1. Duplicate and obviously unrelated articles were eliminated by a single reviewer (Z.C.). Abstracts of the remaining articles were examined independently by three reviewers (Z.C., Z.Y., and X.L.) to determine whether the full-text article should be obtained. Articles published in English or Chinese language, peer-reviewed journals that assessed the relationship between germline polymorphic variants and major outcomes of interest were included. We selected related studies using following inclusion criteria:

(1) Studies should contain the information to estimate relative risks (i.e., Odds ratios [ORs], hazard ratios [HRs]) and 95% confidence intervals (CIs) for prognostic effect of lung cancer.

(2). The cases in studies should be advanced, recurrent, or metastatic lung cancer patients treated by any of the platinum drugs.

(3) Cancer patients should be histologically or pathologically confirmed.

(4) SNPs in *XRCC1 *gene should be genotyped.

(5) The studies with cell line would be excluded.

According to these criteria, a total of 37 articles were selected. Five studies were excluded because data that overlapped from the same study group were published. Three studies were excluded because critical missing information was not obtained. As the number of relevant studies was not enough, two studies with SNPs rs25489, rs2854510 and rs1001581 in *XRCC1 *gene, two studies with *XRCC1 *T-77C, and two studies with toxicity were also excluded. One study only about XRCC1 protein was also excluded. Finally we summarized the results of 22 articles for our systematic review (Table 1).

**Table 1:** Studies on prognosis and *XRCC1 *Arg194Trp and Arg399Gln polymorphisms included in the meta-analysis

Study	Year	Country	Ethnicity	**Case No**.	Stage	Outcome	SNPs
Gao et al. [24]	2006	China	Asian	57	II-IV	TR	*XRCC1 *Arg194Trp and Arg399Gln
Ding et al. [25]	2010	China	Asian	54	IIIB-IV	TR	*XRCC1 *Arg399Gln
Yuan et al. [26]	2006	China	Asian	200	advanced	TR	*XRCC1 *Arg194Trp
Shi et al. [27]	2006	China	Asian	112	II-IV	TR	*XRCC1 *Arg194Trp and Arg399Gln
Qiu et al. [28]	2009	China	Asian	107	IIIA/B-IV	TR	*XRCC1 *T-77C and Arg194Trp
Sun et al. [18]	2009	China	Asian	87	IV	TR	*XRCC1 *Arg194Trp and Arg399Gln
Song et al. [19]	2007	China	Asian	97	IIIB-IV	TR	*XRCC1 *Arg194Trp and Arg399Gln
Wang et al. [20]	2004	China	Asian	105	IIIB-IV	TR	*XRCC1 *Arg194Trp and Arg399Gln
Hong et al. [21]	2009	China	Asian	164	III-IV	TR	*XRCC1 *Arg194Trp and Arg399Gln
Jin et al. [22]	2006	China	Asian	162	IIIB-IV	TR	*XRCC1 *Arg194Trp
Qian et al. [23]	2010	China	Asian	107	IIIA/B-IV	TR	*XRCC1 *Arg399Gln
Cheng et al. [29]	2011	China	Asian	120	advanced	TR	*XRCC1 *Arg399Gln
Giachino et al. [30]	2007	Italy	Caucasian	248	IIIA/B-IV	TR/OS	*XRCC1 *Arg399Gln
Butkiewicz et al. [31]	2010	Poland	Caucasian	162	I, II or IIIA	OS	*XRCC1 *Arg399Gln
de las Penas R. et al. [32]	2006	Spain	Caucasian	135	IIIB-IV	OS	*XRCC1 *Arg399Gln
Yin et al. [33]	2009	China	Asian	257	I- IV	OS	*XRCC1 *Arg399Gln
Sreeja et al. [34]	2008	India	Caucasian	211	I- IV	OS	*XRCC1 *Arg399Gln
Gurubhagavatula et al. [35]	2004	USA	Caucasian	103	IIIA/B-IV	OS	*XRCC1 *Arg399Gln
Kalikaki et al. [36]	2009	Greece	Caucasian	119	IIIA/B-IV	TR/OS	*XRCC1 *Arg399Gln
Liu et al. [37]	2008	China	Asian	53	I- IV	OS/PFS	*XRCC1 *Arg399Gln
Han et al. [38]	2011	Korea	Asian	158	IIIB-IV	OS/PFS	*XRCC1 *Arg399Gln
Yao et al. [39]	2009	China	Asian	108	IIIA/B-IV	OS	*XRCC1 *Arg399Gln

TR: objective response

OS: overall survival

PFS: progression-free survival

The following information were extracted and coded by using standard form: year of publication, country/region of study, ethnicity, case number, cancer stage of samples, outcomes and SNPs included in each study.

### Statistical methods

We abstracted ORs, HRs and 95%CIs from all studies. If such data were missing from some studies, we calculated the ORs and 95%CIs for objective response vs. no response after platinum-based chemotherapy [complete response (CR) + partial response (PR) vs. progressive disease (PD) + stable disease (SD)] using the WHO criteria or the Response Evaluation Criteria in Solid Tumors (RECST). We then investigated the between-study heterogeneity by the Cochran's Q test (a significance level of P < 0.05) and quantified by I^2^. To obtain summary statistics for HRs of overall survival or ORs of chemotherapy response, we performed initial analyses with a fixed-effect model, and confirmatory analyses with a random-effect model if there was significant heterogeneity. The effect of publication bias was examined by inverted funnel plots and the Begg's test. All of P values were two-sided and all analyses were performed using the Stata software version 11.0 (Stata Corp, College station, TX).

## Results

The data pool was composed of 22 studies. Sample sizes ranged from 53 to 257 (median 115.5), including 294 stage I/II and 2632 stage III/IV (total 2926) lung cancer patients (Table 1).

### *XRCC1 *Arg194Trp

Since the study on the association between *XRCC1 *Arg194Trp polymorphism with PFS or OS was too few to be applied in the present meta-analysis, we only analyzed the association of *XRCC1 *Arg194Trp polymorphism and objective response in lung cancer patients.

**Figure 2:**
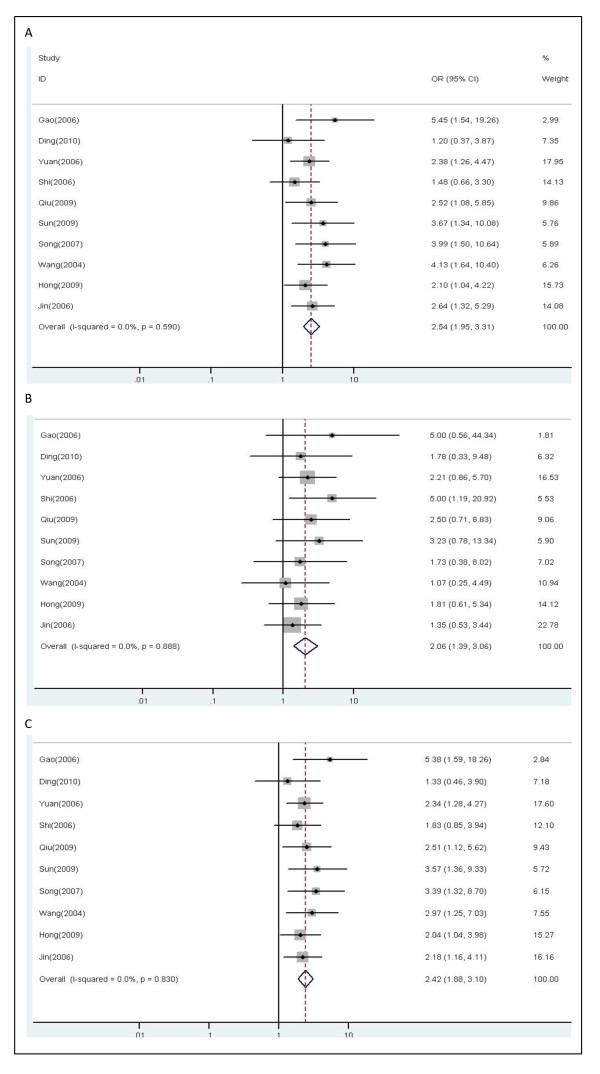
**Forest plot of objective response in lung cancer patients treated with chemotherapy by *XRCC1 *Arg194Trp polymorphism**: (A) C/T vs.C/C; (B) T/T vs. C/C; (C) C/T+T/T vs. C/C.

Ten studies including 1145 patients were eligible for final analysis. The patients bearing the favorable *XRCC1 *194 genotypes (C/T, T/T) were more likely to respond to platinum-based chemotherapy compared with those with the unfavorable genotype (C/C) (C/T vs. C/C: OR, 2.54; 95% CI, 1.95-3.31; P = 0.590 for heterogeneity, I^2 ^= 0.0%; T/T vs. C/C: OR, 2.06; 95% CI, 1.39-3.06; P = 0.888 for heterogeneity, I^2 ^= 0.0%) (Figure 2). In the dominant model, the minor variant T allele also significantly influenced the objective response in all patients (C/T+T/T vs. C/C: OR, 2.42; 95% CI, 1.88-3.10; P = 0.830 for heterogeneity, I^2 ^= 0.0%). No publication bias was detected by either the inverted funnel plot or Begg's test (data not shown).

### *XRCC1 *Arg399Gln

#### Objective response

**Figure 3:**
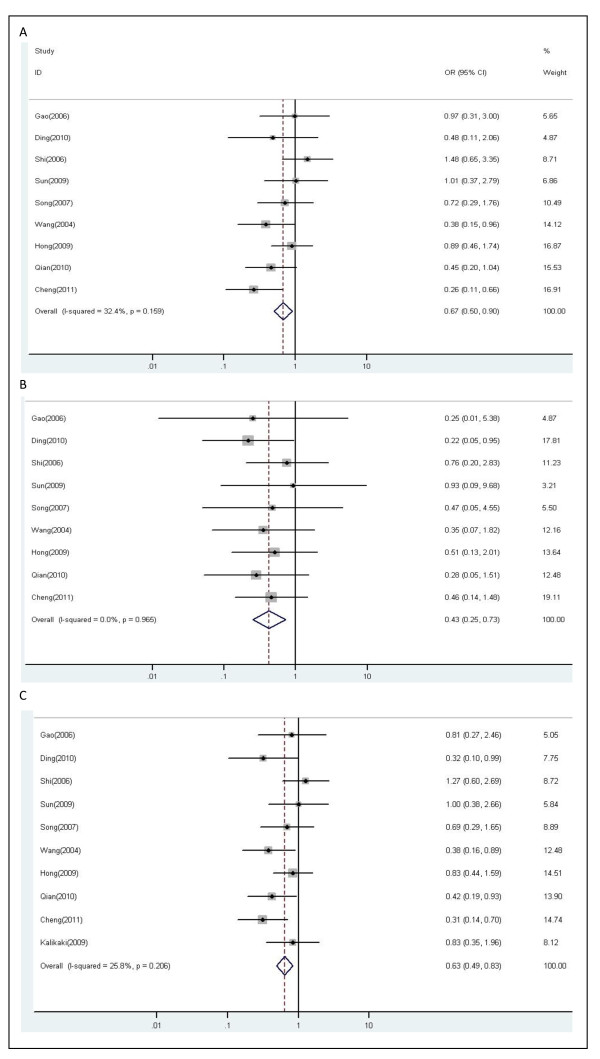
**Forest plot of objective response in lung cancer patients treated with chemotherapy by *XRCC1 *Arg399Gln polymorphism**: (A) G/A vs.G/G; (B) A/A vs. G/G; (C) G/A+A/A vs.G/G.

There were ten studies, altogether 1021 patients, qualified for final analysis. As shown in Figure 3, the *XRCC1 *Arg399Gln polymorphism was significantly associated with response to platinum-based chemotherapy. In the dominant model, the G/A and A/A genotypes of *XRCC1 *399 polymorphism were statistically significantly associated with unfavorable objective response in patients treated with platinum-based chemotherapy (G/A vs. G/G: OR, 0.67; 95% CI, 0.50-0.90; P = 0.159 for heterogeneity, I^2 ^= 32.4%; A/A vs. G/G: OR, 0.43; 95% CI, 0.25-0.73; P = 0.965 for heterogeneity, I^2 ^= 0.0%). The patients with either one or two minor variant A allele showed poorer objective response in all patients (G/A+A/A vs. G/G: OR, 0.63; 95% CI, 0.49-0.83; P = 0.206 for heterogeneity, I^2 ^= 25.8%) (Figure 3). No publication bias was indicated according to the results of the inverted funnel plot and Begg's test (data not shown).

#### Overall survival

##### G/A genotype and A/A genotype

**Figure 4:**
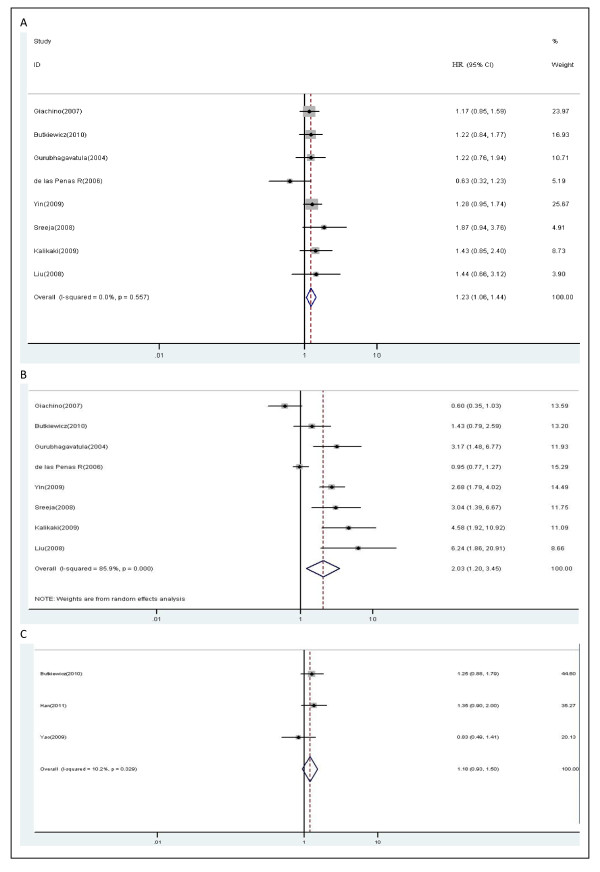
**Forest plot of overall survival in lung cancer patients treated with chemotherapy by *XRCC1 *Arg399Gln polymorphism**: (A) G/A vs.G/G; (B) A/A vs. G/G; (C) G/A+A/A vs.G/G.

A total of eight studies (1288 patients) were included in this part of analysis. Variant genotypes of *XRCC1 *399 polymorphism were associated with higher risks of death for lung cancer patients (Figure 4). With the G/G genotype at *XRCC1 *Arg399Gln being the reference, the HR for G/A genotype was 1.23 compared to 2.03 in the A/A genotype (G/A vs. G/G: HR, 1.23; 95% CI, 1.06-1.44; P = 0.557 for heterogeneity, I^2 ^= 0.0%; A/A vs. G/G: HR, 2.03; 95% CI, 1.20-3.45; P = 0.000 for heterogeneity, I^2 ^= 85.9%). No publication bias was found through either the funnel plot or Begg's test (data not shown).

##### Minor variant A allele

Three studies including 428 patients were eligible for analyzing the relationship between alleles of *XRCC1 *399 polymorphism and overall survival. In the dominant model, there was no evidence for an influence of *XRCC1 *399 A allele on overall survival of lung cancer patients (G/A and A/A vs. G/G: HR, 1.18; 95% CI, 0.93-1.50; P = 0.329 for heterogeneity, I^2 ^= 10.2%) (Figure 4C).

### Interaction of *XRCC1 *Arg194Trp and Arg399Gln polymorphisms on the objective response

**Figure 5:**
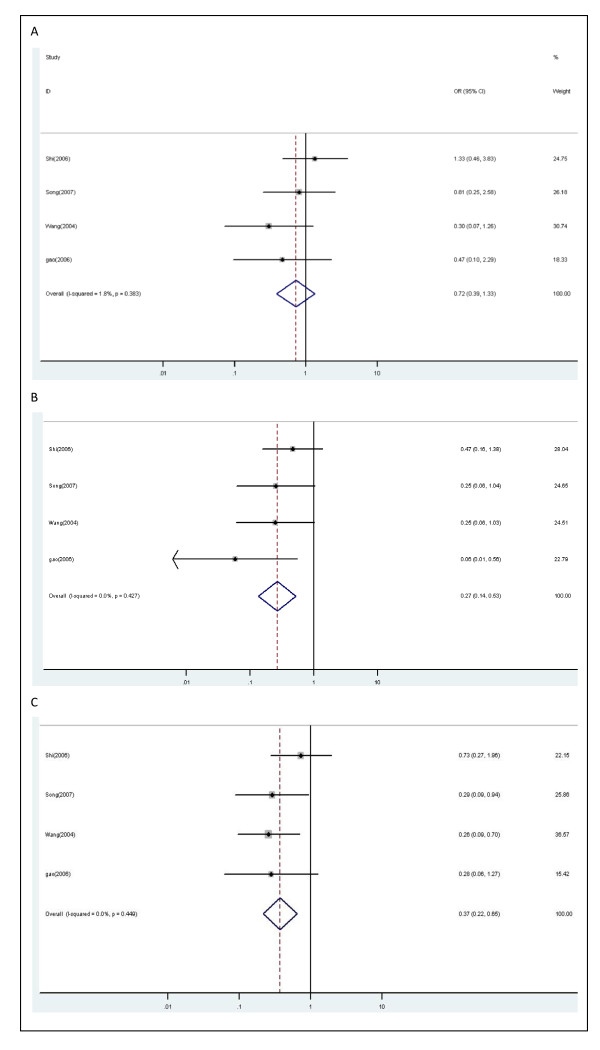
**Forest plot of objective response in lung cancer patients treated with chemotherapy by combinations of *XRCC1 *Arg194Trp and *XRCC1 *Arg399Gln polymorphisms**: (A) combination of 194 C/T+T/T and 399 G/A+A/A vs. combination of 194 C/T+T/T and 399 G/G; (B) combination of 194 C/C and 399 G/G vs. combination of 194 C/T+T/T and 399 G/G; (C) combination of 194 C/C and 399 G/A+A/A vs. combination of 194 C/T+T/T and 399 G/G.

Four studies including 371 patients were used to analyze the relationship of the combinations between *XRCC1 *Arg194Trp and Arg399Gln genotypes with the objective response in lung cancer patients. According to above results in the present study and the results in available studies analyzing the combination of SNPs, we defined combination of *XRCC1 *194 C/T+T/T genotype and *XRCC1 *399 G/G genotype as reference group. Combination of XRCC1 194 C/T+T/T genotype and XRCC1 399 G/A+A/A genotype were not significantly associated with objective response (OR, 0.72; 95% CI, 0.39-1.33; P = 0.383 for heterogeneity, I^2 ^= 1.8%). Combination of XRCC1 194 C/C genotype and XRCC1 399 G/G genotype were significantly associated with objective response (OR, 0.27; 95% CI, 0.14-0.53; P = 0.427 for heterogeneity, I^2 ^= 0.0%). Combination of XRCC1 194 C/C genotype and XRCC1 399 G/A+A/A genotype were significantly associated with objective response in all patients (OR, 0.37; 95% CI, 0.22-0.65; P = 0.449 for heterogeneity, I^2 ^= 0.0%) (Figure 5).

Table 2 summarized the association between XRCC1 Arg194Trp and Arg399Gln polymorphisms with response and overall survival in fixed-effect models and random-effect models, respectively. P values for the between-study heterogeneity were also listed in Table 2.

**Table 2:** Association between *XRCC1 *Arg194Trp and Arg399Gln polymorphisms with objective response and overall survival

XRCC1	Objective response				Overall survival			
		
Arg194Trp	Study	Fixed effect	Random effect	Phet*	Study	Fixed effect	Random effect	Phet*
C/T vs. C/C	10	2.54[1.95,3.31]	2.54[1.95,3.32]	0.590				
T/T vs. C/C	10	2.06[1.39,3.06]	2.06[1.38,3.08]	0.888				
T/T+C/T vs. C/C	10	2.42[1.88,3.10]	2.41[1.87,3.10]	0.830				
XRCC1	Objective response				Overall survival			
		
Arg399Gln	Study	Fixed effect	Random effect	Phet*	Study	Fixed effect	Random effect	Phet*

G/A vs. G/G	9	0.67[0.50,0.90]	0.67[0.46,0.97]	0.159	8	1.23[1.06,1.44]	1.23[1.06,1.44]	0.557
A/A vs. G/G	9	0.43[0.25,0.73]	0.43[0.25,0.84]	0.965	8	1.41[1.19,1.67]	2.03[1.20,3.45]	0.000
A/A+G/A vs. G/G	10	0.63[0.49,0.83]	0.63[0.46,0.86]	0.206	3	1.18[0.93,1.50]	1.18[0.92,1.51]	0.329
Interaction	Objective response				Overall survival			
		
	Study	Fixed effect	Random effect	Phet*	Study	Fixed effect	Random effect	Phet*

c2 vs. c1	4	0.72[0.39,1.33]	0.73[0.39,1.38]	0.383				
c3 vs. c1	4	0.27[0.14,0.53]	0.28[0.14,0.57]	0.427				
c4 vs. c1	4	0.37[0.22,0.65]	0.38[0.21,0.67]	0.449				

Combinations of *XRCC1 *Arg194Trp and *XRCC1 *Arg399Gln polymorphisms: c1, combination of 194 C/T + T/T and 399 G/G; c2, combination of 194 C/T + T/T and 399 G/A + A/A; c3: combination of 194 C/C and 399 G/G; c4, combination of 194 C/C and 399 G/A + A/A.

*Phet: P values for the between-study heterogeneity.

## Discussion

There have been previous studies investigating possible associations between *XRCC1 *SNPs and chemotherapy outcomes or overall survival of lung cancer [18-39]. However these original results are inconsistent and until now the lack of systematic review evaluation failed to give further insights on this issue. We showed, by an extensive quantitative and systematic review of published reports, that *XRCC1 *194 C/T and *XRCC1 *399 G/A SNPs were associated with objective response and *XRCC1 *399 G/A genotype and A/A genotype could influence overall survival of lung cancer patients. Furthermore, interaction analysis suggested that compared with the patients carrying C/T+T/T genotype at *XRCC1 *194 and G/G genotype at *XRCC1 *399, the patients carrying 194 C/C and 399 G/A+A/A or 194 C/C and 399 G/G genotype showed much worse objective response.

Platinum agents are activated intracellularly to form reactive platinum complexes that bind to DNA, thereby inducing intrastrand and interstrand DNA cross-links, as well as DNA-protein cross-links. These platinum-induced DNA and protein effects result in apoptosis and cell growth inhibition. There are several signal transduction pathways involved in this process to exert antitumor effects, among which DNA damage recognition and repair are important. Cancer cells may be able to resist against the platinum-based chemotherapy when its DNA repair ability is enhanced to remove those DNA adducts caused by platinum agents. There is evidence that lung cancer patients with a lower DNA repair capacity had an increased overall survival after the first-line platinum-based chemotherapy [7].

Genetic polymorphisms can contribute directly to the variety in phenotypic drug sensitivity by modifying functions of the related genes. SNPs as either prognostic or predictive biomarkers have many advantages, especially in the advanced cancer setting. Firstly, it is relatively easy to obtain the human specimen for detecting SNP. Secondly, the method to detect SNP is precise and practical. Finally, some biomarkers are detected by specialized and mostly body-harmed methods; otherwise SNP detecting could avoid these problems.

The XRCC1 protein is considered to play an important role in DNA damage repair. *XRCC1 *Arg194Trp and Arg399Gln polymorphisms were the commonest one among more than 60 validated SNPs in *XRCC1 *gene and showed no major variations by ethnicity [40]. *XRCC1 *SNPs have been reported to be associated with an altered DNA repair activity [14,15]. Previous reports have also suggested that *XRCC1 *polymorphisms might be risk factors for the development of lung cancer [41-46] and promising predictive or prognostic makers for lung cancer patients [33-35,47]. Therefore, functional SNPs in *XRCC1 *gene may relate with platinum sensitivity and have prognostic values among lung cancer patients. With a pooled dataset of 2926 patients, we performed a comprehensive and systematic evaluation of clinical outcomes by objective response and overall survival. We are delighted to find the statistically significant association between *XRCC1 *SNPs with objective response and overall survival of lung cancer, which were not significant in previous original reports.

Meanwhile, the interaction between these two SNPs of *XRCC1 *gene in the objective response was analyzed for the first time. It is important to analyze multiple SNPs and their interaction to find more reliable prognostic or predictive biomarkers, because it is hard to predict complex clinical outcomes of lung cancer patients by using only one SNP. Although there were only four original studies included in the present interaction analysis, the results are valuable. The results of interaction analysis showed that the patients carrying 194 C/C and 399 G/A+A/A or 194 C/C and 399 G/G genotype showed worse objective response, suggesting C allele at *XRCC1 *194 may be more significantly associated with poorer objective response than A allele at *XRCC1 *399. The interaction between these two SNPs in objective response of lung cancer patients was not found. The reason may be the interaction between these two SNPs does not exist at all, or false positive report probability (FPRP) is too higher due to the small sample size. So future studies with large sample size on analyzing multiple genes may be necessary to explain the SNPs interaction.

There are three important questions should be addressed in interpreting the results of meta-analysis: (1) were all relevant studies included in the analysis? This is an important question but difficult to assess. We made every effort to search and collect studies that were sufficient to estimate impact of *XRCC1 *SNPs on clinical outcomes and survival of lung cancer as of June 2011; (2) was there heterogeneity in the study? The differences in study population including age, gender, smoking status, cancer histopathology type and cancer stage, in chemotherapy schedule of patients, and in measurement of confounding factors and others may result in study heterogeneity. In the present study, the between-study heterogeneity was analyzed by the Cochran's Q test (P < 0.05) and quantified by I^2^. Indeed, heterogeneous effects were observed in one subset in the present study. However, we could not separate studies further to obtain homogeneous groups because there was no information on confounding factors. So we performed initial analyses with a fixed-effect model and confirmatory analyses with random-effect model, if there was significant heterogeneity. At last we found the results were similar between fixed-effect model and random-effect model. On the other hand, when the studies, which are possible source of heterogeneity, were excluded from the analyses, there were similar results observed, which also showed that the heterogeneity did not appear to impact significantly on the results of our analyses; (3) publication bias is an important problem in meta-analysis and occurs if scientific studies with negative or null results fail to get published; this can happen due to bias in submitting, reviewing, accepting, publishing or aggregating scientific literature that fails to show positive results on a particular topic; and it could make scientific literature unrepresentative of the actual research studies [48]. In our results, no evidence of publication bias was found using standard tests such as inverted funnel and the Begg's test. However, we should know that these methodologies did not completely exclude biases, because there might have been rejection or even non-submission of negative results.

Despite our efforts in performing a comprehensive analysis, limitations of our meta-analysis need to be stated. First, most of the included studies differed in their study designs, such as subject selection, chemotherapeutic protocol et al. The patients in some studies also had surgery or other treatment such as radiotherapy in addition to the chemotherapy. All these confounding factors may influence the homogeneity between studies. Stratified analyses by possible confounding factors such as smoking history, cancer histology, and treatment method, might be able to reduce the heterogeneity as stated above. However, few of these studies reported SNP genotype distribution by subgroups so such analyses were impossible to implement. Second, our analyses mostly used unadjusted estimates, because not all published studies stated adjusted estimates, or when they stated, the estimates in different studies were adjusted by different possible confounding factors. However, when the available adjusted estimates were used in our analyses, the conclusions have not been significantly changed (data not shown). Third, we did not analyze the association between *XRCC1 *SNPs and progression-free survival because there are only two studies involving progression-free survival. Fourth, the relationship between *XRCC1 *SNPs and platinum-based chemotherapy toxicities was not able to be analyzed in the present study, because few studies provided the related results.

In conclusion, our meta-analysis suggested that *XRCC1 *Arg194Trp and Arg399Gln polymorphisms may be associated with overall survival and response to platinum-based chemotherapy in lung cancer patients. Lung cancer patients with *XRCC1 *194 T allele or *XRCC1 *399 G allele may benefit from platinum-based chemotherapy. However, to address these issues, future prospective studies with large sample size and even randomized clinical trials may be necessary. Besides, efforts on analyzing multiple genes to find more reliable prognostic or predictive biomarkers and even on studying gene-environment interaction should be made, because it is hard to predict complex clinical outcomes of lung cancer patients by using only single gene.

## Conclusions

Genetic polymorphisms in *XRCC1 *gene might be associated with overall survival and response to platinum-based chemotherapy in lung cancer patients.

## Competing interests

The authors declare that they have no competing interests.

## Authors' contributions

ZC participated in extracting the data, performing the statistical analysis and drafting the manuscript. ZY participated in selecting study, extracting data and drafting the manuscript. XL and WW collected and extracted the data. PG has been involved in revising the manuscript critically for important intellectual content. BZ conceived of the study and participated in drafting the manuscript. All authors read and approved the final manuscript.

## Pre-publication history

The pre-publication history for this paper can be accessed here:

http://www.biomedcentral.com/1471-2407/12/71/prepub
